# Minimal Cut Sets and the Use of Failure Modes in Metabolic Networks

**DOI:** 10.3390/metabo2030567

**Published:** 2012-09-11

**Authors:** Sangaalofa T. Clark, Wynand S. Verwoerd

**Affiliations:** Center for Advanced Computational Solutions (C-fACS), Deptment of Wine, Food & Molecular Biosciences, Faculty of Ag & Life Sciences, P O Box 84, Lincoln University, Lincoln 7647, Christchurch, New Zealand

**Keywords:** minimal cut sets, elementary modes, metabolic networks

## Abstract

A minimal cut set is a minimal set of reactions whose inactivation would guarantee a failure in a certain network function or functions. Minimal cut sets (MCSs) were initially developed from the metabolic pathway analysis method (MPA) of elementary modes (EMs); they provide a way of identifying target genes for eliminating a certain objective function from a holistic perspective that takes into account the structure of the whole metabolic network. The concept of MCSs is fairly new and still being explored and developed; the initial concept has developed into a generalized form and its similarity to other network characterizations are discussed. MCSs can be used in conjunction with other constraints-based methods to get a better understanding of the capability of metabolic networks and the interrelationship between metabolites and enzymes/genes. The concept could play an important role in systems biology by contributing to fields such as metabolic and genetic engineering where it could assist in finding ways of producing industrially relevant compounds from renewable resources, not only for economical, but also for sustainability, reasons.

## 1. Introduction

Minimal cut sets (MCSs) have been developed from elementary modes (EMs) [1,2,3,4], a metabolic pathway analysis (MPA) [5,6,7] method that uses convex analysis [8,9] to identify all possible and feasible metabolic routes for a given network at steady state. A review of the history of EMs can be seen in [10].

This review focuses on MCSs which, together with EMs, form dual representations of metabolic networks with both being able to be converted into each other [11]. The MCSs approach identifies target genes for eliminating a certain objective function; it adds to the increasing importance of MPA methods [5,6,7], and the capacity to employ metabolic engineering and biological systems to produce industrially relevant compounds from renewable resources, by providing a means of finding suitable targets for repressing undesirable metabolic functions. 

MCSs can be considered the smallest “failure modes” in a system; they were first introduced in 2004 by S. Klamt and Gilles [12], motivated by their desire to gain deeper insight into the functionality and capability of an organism by further analyzing the structure of its metabolic network. In particular, they looked at how potential failure modes in a metabolic network could render the network structurally incapable of performing certain functions. They subsequently developed an algorithm, which was later generalized [11], for computing MCSs and identifying crucial parts in the network structure and suitable targets for repressing undesired metabolic functions. The calculation and analysis of MCSs and EMs are features of the CellNetAnalyzer program [13]. 

## 2. Defining Minimal Cut Sets

S. Klamt and E.D. Gilles [12] defined MCSs as follows:

*“We call a set of reactions a cut set (with respect to a defined objective reaction) if after the removal of these reactions from the network no feasible balanced flux distribution involves the objective reaction”*; and *“A cut set C (related to a defined objective reaction) is a minimal cut set (MCS) if no proper subset of C is a cut set.”*

In effect, an MCS (with respect to an objective reaction) constitutes the minimal set of reactions whose removal from the network prevents any feasible balanced flux distribution involving the objective reaction; MCSs are the minimal hitting sets of the target EMs [14] or the minimal sets of knockouts that disable the operation of a specified set of target elementary modes [15].

In terms of the network structure, a continued operation of the objective reaction would not be physiologically possible because it would lead to the depletion or accumulation of metabolite pools and the system would not be able to achieve steady state.

### 2.1. The Initial Concept of MCSs

The algorithm for calculating MCSs was developed by S. Klamt and E.D. Gilles [12] and operates on EMs [1,2,4]. In fact, EMs and MCSs complement each other, as will be discussed later on. 

The theory behind the use of EMs [1,2,4] for calculating MCSs is the fact that an EM is minimal, thus non-decomposable in terms of the reactions (enzymes) utilized; removing a reaction from an EM results in the system not being able to achieve steady state with the remaining reactions of the EM. So, if the objective reaction is identified for the network function of interest, and EMs are calculated for it, the MCSs would be the reactions that, if taken out, would result in the system not being able to achieve steady state with the remaining reactions in these particular EMs, *i.e.*, cause the dysfunction of the system with respect to the objective reaction, so the corresponding network function is repressed.

MCSs can be used for studying the fragility of a network structure and identifying suitable targets for metabolic functionalities. For example, we have used MCSs [16] to study the functionalities of anthocyanin related genes in flowering plants.

### 2.2. Example Network to Illustrate MCSs

**Figure 1:**
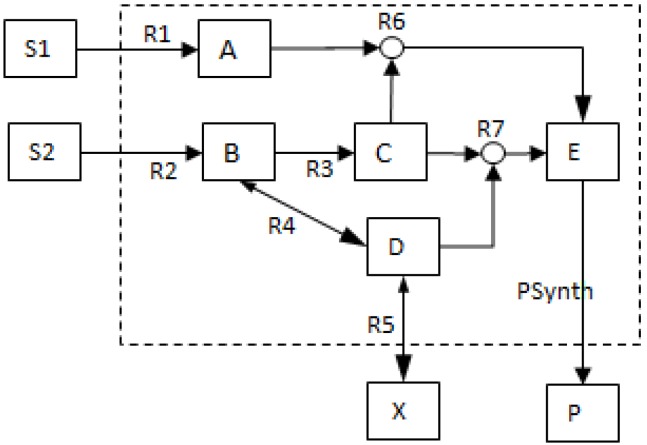
Network layout for an example network (*NetEx*) discussed in [11].

To illustrate the MCS concept, consider the example network (*NetEx*) used in [11] and shown in Figure 1 below. The characteristics and hypergraphical nature of the network are important in defining its MCSs.

Features to Note about *NetEx*

- The network consists of five internal metabolites and eight reactions, of which R4 and R5 are reversible;
- Reactions crossing the system boundaries are coming from/leading to buffered/buffer metabolites.

Consider the case where the synthesis of product *P* is of particular interest; then, the reaction *PSynth* is the objective reaction in the same context as a target reaction [17], whereby all flux vectors with a non-zero flux through reaction *PSynth* are of importance. 

**Figure 2:**
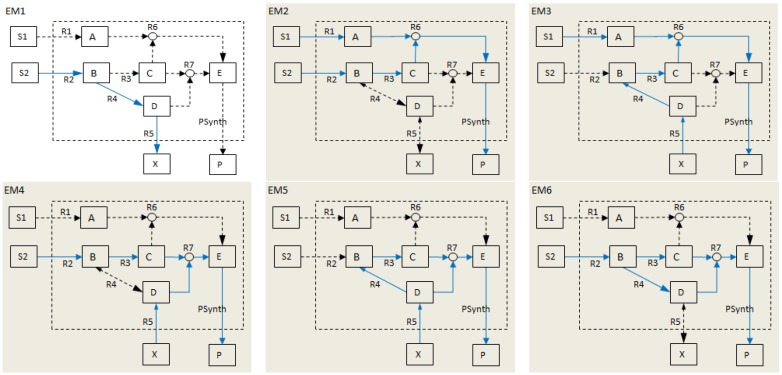
Elementary modes (EMs) for *NetEx*. The EMs are represented by the solid blue arrows. Adapted from [11].

The first step would be to determine the qualitatively distinct possible ways of producing *P*; this is equivalent to calculating EMs as illustrated in Figure 2 below.

As shown in the above figure, there are six EMs for *NetEx*, five of which involve the *PSynth* reaction (highlighted networks). In order to eliminate the production of *P*, all the EMs that involve *PSynth* need to be blocked. By definition, an EM is blocked by removing any of its constituent reactions, therefore, any combination of reactions, one taken from each EM, forms a cut set that disables flux through the EMs. For our network example, *NetEx*, a MCS for the objective reaction, *PSynth*, is a set of reactions whose knockout blocks the five EMs involving *PSynth*, thus disabling flux through *PSynth* at steady state. 

### 2.3. Other Definitions

The notion of MCSs does exist in other theories and research areas, particularly in relation to risk analysis. In developing the algorithm for MCSs, S. Klamt and E.D. Gilles [12] looked at previous similar definitions of MCSs that existed in other areas at the time. These included fault trees and graph theory which shall be discussed here; other similar concepts are looked at later in Section 5.

#### 2.3.1. Fault Trees

Fault Trees are non-recursive Boolean networks studied in reliability and risk assessment of industrial systems [18,19], which have similar definitions of MCSs. The Fault Tree diagrams use logic block diagrams to display the state of a system (top event) in terms of the states of its components (basic events). The basic events are ‘entries’ at the lowest level which form the leaves of the tree; intermediate events are those produced by binary operations (e.g., AND, OR, XOR) of other events, and the top event, representing a usually undesired system failure, is at the top of the Fault Tree. 

**Figure 3:**
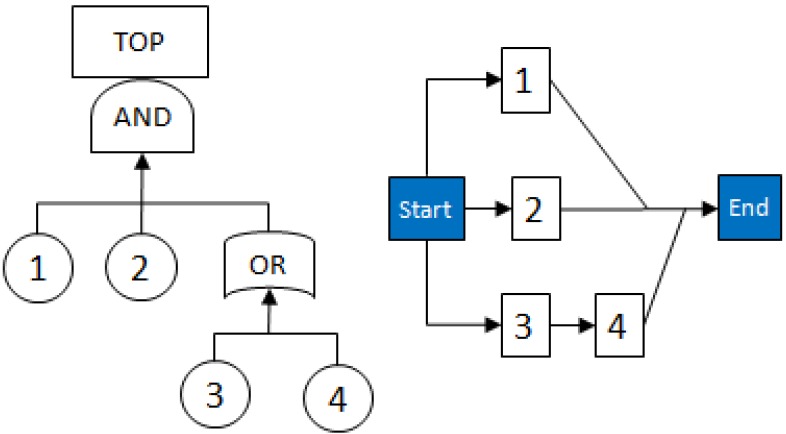
Example of a Fault Tree with equivalent Reliability Block Diagram (RBD).

An example of a Fault Tree can be seen in the left hand graph of Figure 3 below. The right hand side graph is a Reliability Block Diagram (RBD) version of the Fault Tree. RBDs inversely represent Fault Trees: in RBDs one is working in the "success space" and thus looks at system success combinations, while in a Fault Tree one is working in the "failure space" and looks at system failure combinations.

**Figure 4:**
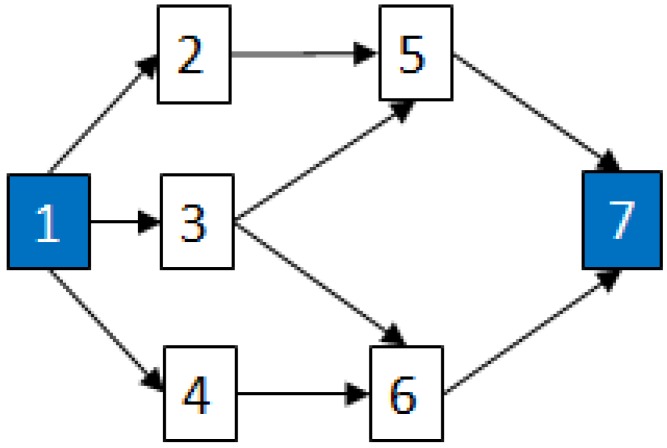
RBD of an example network system.

**Figure 5:**
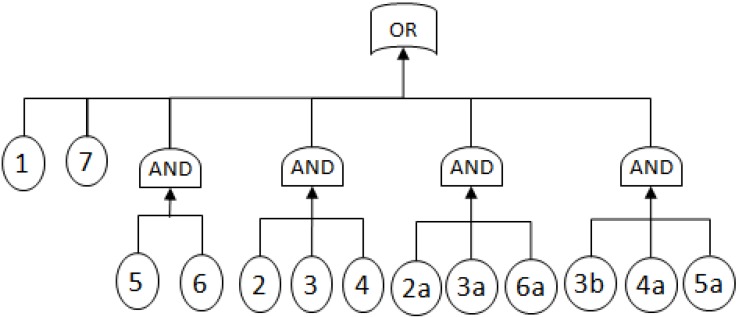
Equivalent Fault Tree of RBD in Figure 4. Blocks 2a-6a, 3b are duplicates of their corresponding blocks.

MCSs [20] for complex RBDs and Fault Trees are used to estimate their reliability. MCSs can also be used to convert a complex diagram/system into a Fault Tree by constructing the RBD of a system, determining the MCSs and then using them to construct the Fault Tree. For example, consider an example system whose RBD is shown in Figure 4 below:

- MCSs obtained from the RBD are: {1}, {7}, {5,6}, {2,3,4}, {2,3,6} and {3,4,5};
- The Fault Tree is constructed by connecting the MCSs using the OR gate. Within each set that contains multiple blocks, the multiple blocks are connected with an AND gate. The equivalent Fault Tree is shown in Figure 5 below:

More about Fault Trees and RBDs and the software used in reliability engineering and related fields can be seen in [21,22]. 

The difference between MCSs in Fault Trees and those in metabolic networks is that unlike RBDs, there is no definite knowledge of which combinations of the removed reactions would cause the failure of the objective reaction So, Fault Tree algorithms cannot be used to determine MCSs in metabolic networks.

#### 2.3.2. Graph Theory

**Figure 6:**
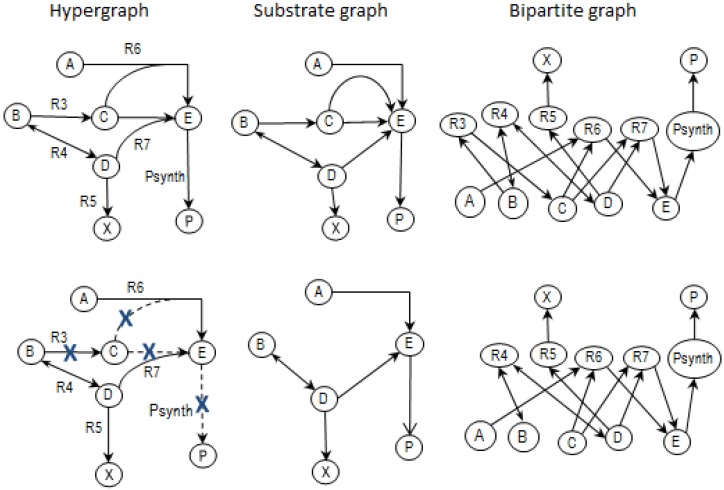
Hypergraph showing reactant and product nodes of R6 and R7 of *NetEx* with corresponding versions of substrate and bipartite graphs. In the lower hypergraph, removing R3 means no C is formed and a consequential removal of R6 and R7, which means that *PSynth* cannot proceed.

Another similar definition of MCSs exists in graph theory [23] where cut sets serve to disconnect a graph. However, the definitions would have different results because, in addition to the stoichiometric relations that need to be satisfied, metabolic network MCSs also need to take into account the hypergraphical nature of the metabolic networks where an edge (reaction) can link reactant nodes with product nodes. For instance, in the example network *ExNet*, reactions R6 and R7 have 2:1 (reactants:product) relationships (hypergraph in Figure 6 below) with compound C being involved in both reactions; substrate and bipartite graphs only allow 1:1 (reactant:product) relations as illustrated in the corresponding *substrate* and *bipartite* versions shown to the right of the hypergraph in the top row of Figure 6 below.

If reaction R3 is eliminated as shown in the second row of Figure 6 above, product P in the hypergraph cannot be formed: you cannot get from A or B to P. However, you can still get to P from both A or B in the substrate and bipartite graphs so the resulting MCSs of the hypergraph (Table 1) will be different from that of the other graphs.

### 2.4. Determining MCSs

Referring to the example network *NetEx* in Figure 1, the MCSs for the objective reaction, *PSynth*, can be determined as follows:

(1) Calculate EMs [3] in *NetEx* and identify those that start from a buffered metabolite and lead to the formation of metabolite *E* or the objective reaction *PSynth*. Since EMs are non-decomposable, removing one of the reactions from these EM will prevent the system from producing *E* and subsequently achieving the *PSynth*.
  There are six EMs in total, of which five lead to the formation of metabolite *X* and the objective reaction.
(2) Determine how to prevent *PSynth* from taking place, *i.e.* stop the five EMs that involve *PSynth* from being functional. This can be done in various ways e.g. inactivating one or more reactions in the EMs by deleting genes of certain enzymes or other manipulations that inhibit the enzymes. Different numbers and combination of reactions can be removed to eliminate *PSynth*.

The MCSs for a given objective reaction in a large metabolic network, however, cannot be done by a simple examination; an algorithm would be needed to compute the MCSs. The first algorithm was developed by Klamt and Gilles [12] although others have been developed since, to improve on the computational speed and efficiency; these are discussed in Section 3.2.

The MCS Algorithm

The MCS algorithm devised by Klamt and Gilles [12] relies on the fact that:

- any feasible steady-state flux distribution in a given network, expressed by a vector of the net reaction rates, *r*, can be represented by a non-negative linear combination of elementary modes as illustrated in Equation 1 (adapted from [11]):

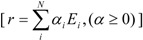
(1)

where *N* is the number of EMs; and

- the removal of reactions from the network results in a new set of EMs constituted by those EMs from the original network that do not involve the deleted reactions [24].

Before MCSs are computed, the set of EMs is split into two disjoint sets:

- the set of target modes (*E^t^*), *i.e.*, all EMs (*e^t,j^*) involving the objective reaction, *t*
- the set of non-target modes (*E^nt^*), *i.e.*, EMs not involving the objective reaction, *nt*

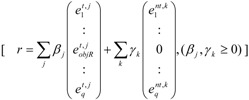
(2)

The right-hand side of Equation 2 above, illustrates, respectively, the set of EMs (e*^,t,j^*) comprising the target modes (*E^t^*) and the set of EMs (e*^nt,k^*) comprising the non-target modes (*E^nt^*) [11]. Since removing a set of MCSs ensures inactivation of all target modes *E^t,j^*, only non-target modes *E^nt,k^* could survive, which means that all remaining flux distributions *r* will show zero flux in the objective reaction, *r_objR_*. 

The pseudocode of the MCS algorithm for calculating MCSs initially developed by S. Klamt and E.D. Gilles is provided in [12] and further modified for the example network, *NetEx*, discussed in [11]. 

**Figure 7:**
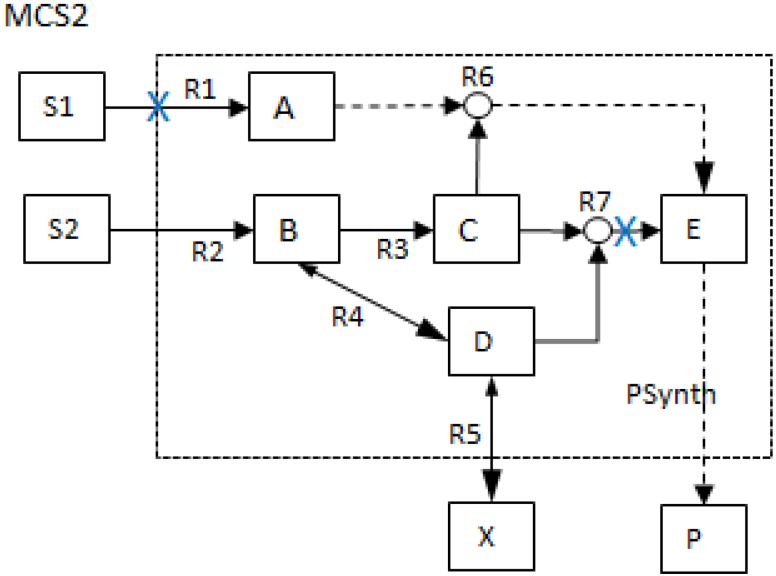
One of the Minimal cut sets (MCSs) for objective reaction *PSynth*: The simultaneous blocking of reactions R1 and R7 will eliminate *PSynth* and block the production of P.

For the *NetEx* network, the algorithm calculates seven MCSs in addition to the trivial MCS (*PSynth* itself). To illustrate, one of the MCSs (MCS2) is shown in Figure 7 below:

The seven MCSs and the corresponding EMs are shown in the first two tables of Table 1.

### 2.5. Generalized Concept of MCSs

S. Klamt, in 2006 [11], redefined the MCS from that of the original concept expressed under 2 earlier, to “*a minimal (irreducible) set of structural interventions (removal of network elements) repressing a certain functionality specified by a deletion task*”. This new definition indicates the key role that the deletion task plays in the difference between the new generalized approach and the initial MCS concept. 

The deletion task can be specified by several Boolean rules that clearly represent and describe, unambiguously, the flux patterns or the functionality to be repressed. This increases the practical applicability of MCSs because they can now be determined for a large variety of complex deletion problems and for inhibiting very special flux patterns instead of just for studying structural fragility and identifying knock-out strategies. 

The refinements and extensions to the initial MCS concept offer a broader range of possible ways in which MCSs can be used to assess, manipulate and design biochemical networks. A comparison of the concept versions is covered later.

### 2.6. Further Refined Concept of MCSs

Further refinement of MCSs has also been undertaken [15] to deal with their limitation of disabling desired functionalities along with the targeted ones. To address this limitation, Hädicke and Klamt [15] generalized MCSs to *Constrained* MCSs (cMCSs) that take into consideration side constraints and allow for a set of desired modes, with a minimum number preserved, to be defined.

This generalization provides a flexibility for cMCSs to be applied to existing methods, for example Minimal Metabolic Functionalities [25,26], OptKnock [27], and RobustKnock [28] can be reformulated as special cases of cMCSs. As demonstrated in [15], the cMCSs approach offers great flexibility in defining and solving knock out problems.

The next section compares the three concepts, to get a better understanding of MCSs and how they have developed.

### 2.7. Comparing MCS Concepts

#### 2.7.1. Same Properties

Some properties between the initial and generalized/ refined concepts of MCSs remain the same. For example:

- there will always be a trivial MCS- the objective reaction itself;
- some reactions such as the biomass synthesis, are actually pseudo-reactions that are not related to a single gene or enzyme and thus cannot be repressed by inhibitions such as gene deletions;
- the definition of the MCSs: each MCS provides a minimal (irreducible) set of deletions or EMs from the set of target modes, that will achieve the elimination of the objective reaction.

#### 2.7.2. Different Properties

- A deletion task *T* is a set of constraints that characterize the stationary flux patterns (reactions) *r* to be repressed while *D*, derived from *T*, characterizes the target modes (EMs) to be targeted by MCSs. As such, *D* (for the target modes) and *T* (for the flux vectors *r*) are, in most cases such as in the earlier MCS concept, identical.
- In the generalized MCS concept, however, the deletion task *D* can either differ from *T* or *T* must be transformed into several *D_i_* that lead to sub-tasks. So, instead of only dealing with a simple deletion task *T* where all non-trivial flux distributions for an objective reaction are blocked, other more complicated deletion tasks and intervention goals are possible.
- In the initial MCS concept, the MCSs are based on EMs, whereas the generalized MCS concept [11] sees EMs and MCSs as dual representations of network functions, which can be converted into each other, *i.e.*, MCSs are EMs in a dual metabolic network [29].
- The generalized MCS concept offers a wider range of capacity to assess, manipulate and design biochemical networks. MCSs are no longer restricted to the removal of reactions as shown in Figure 2 but can also contain network nodes such that more general deletion problems can be tackled. The MCSs that involve the removal of other network parameters besides reactions are shown in the lower two tables (1b and 1c) of Table 1 below.

**Table 1:** Elementary modes and the different types of MCSs of *NetEx* for the objective reaction *PSynth.* Initial MCS concept: 1a): removing reactions only; Generalized MCS concept: 1b) removing metabolites only, and 1c) reactions and metabolites together. Note: a non-zero in the EM cell indicates the reaction occurs in the EM; a “1” in the MCS indicates the reaction constitutes the MCS. Adapted from [11].

Elementary modes EM2-EM6 (grey) involve the objective reaction *PSynth.*													
	R1	R2	R3	R4	R5	R6	R7	*PSynth*	A	B	C	D	E
EM1	0	1	0	1	1	0	0	0	0	1	0	1	0
EM2	1	1	1	0	0	1	0	1	1	1	1	0	1
EM3	1	0	1	−1	−1	1	0	1	1	1	1	1	1
EM4	0	1	1	0	−1	0	1	1	0	1	1	1	1
EM5	0	0	1	−1	−2	0	1	1	0	1	1	1	1
EM6	0	2	1	1	0	0	1	1	0	1	1	1	1
MCSs of *NetEx* for the objective reaction *PSynth*													
1a) Initial concept: MCSs removing reactions only													
MCS0								1					
MCS1			1										
MCS2	1						1						
MCS3						1	1						
MCS4		1		1									
MCS5		1			1								
MCS6	1			1	1								
MCS7				1	1	1							
1b) Generalized concept: Minimal cut sets removing metabolites only													
MCS8										1			
MCS9											1		
MCS10													1
MCS11									1			1	
1c) Generalized concept: Minimal cut sets removing reactions and metabolites													
MCS12							1		1				
MCS13				1	1				1				
MCS14	1											1	
MCS15		1										1	
MCS16						1						1	

From Table 1 we can compare the number of MCSs obtained from removing reactions only (initial MCS concept) or other parameters (generalized concept). The least number of MCSs occurs when removing metabolites (1b), which implies that metabolites are more crucial for the production of *P*; this is evident when we look at the set of EMs which shows three metabolites as essential for *PSynth* compared to one essential reaction. This is because removing a metabolite results in eliminating all the reactions connected to it, thus eliminating the corresponding EMs, so MCSs from deleting metabolites would be more effective. MCSs could also be derived from a combination of reactions and metabolites, although these methods could quickly become computationally challenging [30,31]; computational complexity is discussed later.

*Constrained* MCSs (cMCSs) provide further flexibility by providing the capacity to specify, not only functionalities to be disabled, but also those that need to be preserved; the combination of these desirable and undesirable functionalities are represented by appropriate sets of target EMs and desired EMs. This allows for systematic enumeration of all equivalent gene deletion combinations and subsequently assists in determining intervention problems and robust knockout strategies for coupled product and biomass synthesis.

For example, consider our network example *NetEx* (Figure 1) which has six EMs; say the objective is to suppress the synthesis of P in order to maximise the production of X; the set of target modes would be T= {EM2, EM3, EM4, EM5, EM6} with the eight MCSs as shown in the first set of MCSs in Table 1. The resulting intervention problems are shown in Table 2 below:

**Table 2:** Intervention problems and resulting MCSs for the example network, *NetEx.*

Intervention Problems		Target modes T	Desired modes D1	n1	MCSs
I1)	No synthesis of undesired product P	EM2, EM3, EM4, EM5, EM6			MCS0={ *Psynth*}, MCS1={R3}, MCS2={R1,R7}, MCS3={R6,R7}, MCS4= {R2, R4}, MCS5={R2,R5}, MCS6={R1,R4,R5}, MCS7={R4,R5,R6}
I2)	No synthesis of undesired product P and production of X with maximal yield possible	EM2, EM3, EM4, EM5, EM6	EM1	1	MCS0={ *Psynth*}, MCS1={R3}, MCS2={R1,R7}, MCS3={R6,R7},

The above *NetEx* example is a very simple case and a more comprehensive example can be seen in [15] which describes cMCSs in detail.

## 3. Computational Complexity

Although recent studies [30] have shown that it is easy to check that a given set of reactions constitutes a cut, finding a MCS for a given set of target compounds becomes impractical in large networks. This stems from the fact that finding all EMs that use a particular reaction is nondeterministic polynomial time hard (NP-hard) [32].

### 3.1. Deterministic and Non-Deterministic Polynomial Complexity

In computational complexity theory [33,34], deterministic polynomial (P) and non-deterministic polynomial time (NP) are two classes of decision problems that classify computational problems according to their inherent difficulty in terms of their solvability by a computer. The computation problem can be stated by a set of mathematical instructions consisting of problem instances and solutions to these problem instances. 

A problem is regarded as inherently difficult if its solution requires significant resources, whatever the algorithm used. The theory formalizes this intuition, by introducing mathematical models of computation to study these problems and quantify the amount of resources needed to solve them, such as time and storage. One of the roles of computational complexity theory is to determine the practical limits on what computers can and cannot do and the big *O* notation is useful for analyzing the run time for class P and NP problems.

The big *O* notation can analyze the efficiency of algorithms such as the time (*T*) (or the number of steps) it takes to complete a problem of size *n*. For example the time might be found to be *T(n) = 6n^2^-2n+5*. As n grows large, the *n^2^* term will come to dominate, so that all other terms can be neglected. The coefficients also become irrelevant if *T(n)* is compared to other orders of expression e.g., *n^3^* or *n^4^*; *U(n) = n^3^*, will always exceed *T*(*n*) when *n* gets larger than 6. The number of steps, on the other hand, depends on the details of the machine model on which the algorithm runs, although different types of machines generally vary by only a constant factor in the number of steps needed to execute an algorithm. So the algorithm has order of *n^2^* time complexity denoted by the big *O* as:


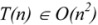
(3)

Note: The following two right-hand side big *O* notations have dramatically different meanings:


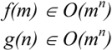
(4)

The first case states that *f(m)* exhibits polynomial growth, while the second, assuming *m > 1*, states that *g(n)* exhibits exponential growth. 

Class P consists of those decision problems whose solution can be obtained using a deterministic algorithm that runs in polynomial time, *i.e*., runs in *O*(*n^k^*)steps for some non-negative integer *k*, where *n* is the input size. A deterministic algorithm only has one choice in each step taken to execute the problem, *i.e*., it would have the same output for every run on the same input instance for the problem.

Class NP consists of those decision problems for which there exists a nondeterministic algorithm that runs in polynomial time with two phases: a) *the guessing phase* where a nondeterministic algorithm is used to generate an arbitrary string of characters that may or may not correspond to a solution of the input, and b) *the verification phase* which uses a deterministic algorithm to check and verify that the generated string is a valid solution or reject it otherwise. Both phases need to be completed in polynomial time (*O*(*n^j^*))where *j* is a non-negative integer. 

The complexity class P is contained in NP, but NP contains many important problems, the hardest of which are called NP-complete problems, for which no polynomial-time algorithms are known for solving them (although they can be verified in polynomial time). The most important open question in complexity theory, the P=NP problem, asks whether such algorithms actually exist for NP-complete, and by corollary, all NP problems. It is widely believed that this is not the case.

The complexity of EMs and MCSs in metabolic networks is covered in [30,31] and are found to be NP-hard. A problem is NP-hard if an algorithm for solving it can be translated into one for solving any NP-problem, so NP-hard means "at least as hard as any NP-problem", although it might, in fact, be harder. 

In addition to finding EMs being NP-hard, it has also been shown [30] that finding cuts of minimum size without computing all EMs is also NP-hard. This doesn’t help from the point of view of genetic intervention where it is desirable to find a MCS of minimum size, thus a need for computational methods to address the problem.

### 3.2. MCS Computational Methods

Four MCS computational methods have been developed since the first two (original and general MCS concepts [11,12]); mainly to improve the computational complexity of obtaining MCSs but they also open MCSs and EMs to a wider area of application:

i) The first method was presented by Imielinski and Belta [35] and considers obtaining cut sets from the computation of sub-EMs which are EMs of a submatrix of the stoichiometry matrix [36]; the submatrix in turn is formed by taking a subset of the rows of the stoichiometry matrix. In other words, the sub-EMs are flux configurations that place only a subset of species in the system at steady state. Because the sub-EMs naturally emerge from the intermediate steps of the tableau algorithm for EM computing [3], it means that the sub-EMs can be obtained from a network of any size, hence overcoming the problem where the metabolic network is too large and complex that it becomes NP-hard to find MCSs. A possible drawback is that there is no guarantee that all the cut sets will be found and their minimality is also not guaranteed so the cut sets would need to be checked for minimality and further reduced to MCSs where necessary. Development of this computational framework is described in detail in [35] as well as its application to a genome scale metabolic model of *E.coli*.
ii) The second method is by Haus *et al.* [14] and involves modifying existing algorithms to develop more efficient methods for computing MCSs. Their first algorithm is a modification of Berge’s algorithm [37] and computes MCSs from EMs, thereby improving on the time and memory required for enumeration; the second algorithm is based on Fredman and Khachiyan [38] and directly computes MCSs from the stoichiometric matrix, with the hypergraph of EMs containing the blocked reactions being generated on the side.
iii) The third method, contributed by Ballerstein *et al.* [29], also determines MCSs directly without knowing EMs. Their computational method is based on a duality framework for metabolic networks where the enumeration of MCSs in the original network is reduced to identifying the EMs in a dual network so both EMs and MCSs can be computed with the same algorithm. They also proposed a generalization of MCSs by allowing the combination of inhomogeneous constraints on reaction rates.
iv) The fourth method includes an approximation algorithm for computing the minimum reaction cut and an improvement for enumerating MCSs, recently proposed by Acuña *et al.* [30]. These emerged from their systematic analysis of the complexity of the MCS concept and EMs, in which it was proved that finding a MCS, finding an EM containing a specified set of reactions, and counting EMs are all NP-hard problems.

The algorithm and enumeration improvement aim to avoid having to compute elementary modes in order to obtain reaction cuts; instead of a MCS that disables too many EMs, it would be desirable to find a MCS that cuts the target reaction but leaves certain reactions intact or as many EMs as possible intact. These types of MCSs are NP-hard. The developments in [30] provide the capacity to analyze the complexity of the underlying computational tasks that would assist in determining which tasks can be tackled.

## 4. Applications of MCSs

MCSs were developed as an extension of the metabolic pathway analysis methods and thus provide a different, if not improved, approach for studying similar network properties. The application of MCSs, as Klamt describes [11] it, can be grouped into two types, depending on how the cuts are provoked in the network:

i) If the cut occurred naturally, e.g., a reaction malfunctioning due to spontaneous mutation, the MCS would serve as an internal failure mode with respect to a certain functionality and could be applied to study structural fragility and robustness on a local and global scale.
ii) If, on the other hand, the cut is a deliberate intervention e.g., gene deletion, enzyme inhibition or RNA interference, then the MCS would be seen as a target set that could, for example, be suitable for blocking metabolic functionalities, and thus have significant potential in metabolic engineering and drug discovery. These applications can be extended to enable the MCSs to be used for assessing/verifying, manipulating and designing biochemical networks.

Because a complex network provides many alternate pathways, there are generally several different MCSs for a single collection of objective reaction(s). All of these MCSs would be effective but their efficiencies would differ. In this respect, MCSs can be used in conjunction with other metabolic pathway methods to gain more information on the structural capability of the network in relation to the objective function. Since the mathematics guarantees that the collection of MCSs is complete, we can use quantitative analysis to compare and investigate the effect that each MCS has on the remaining non-target set of EMs. Along with other different MPA methods, these effects can be utilized in exploring things such as which MCSs would achieve loss-of-function most efficiently and whether this was related to the position of the genes in the pathway. Other investigations could include correlating different MCSs to different structures and/or situations. We could also analyze the properties of the genes concerned and the impact that their suppression would have on other processes in the network. 

The next part looks at areas in which MCSs have been applied.

### 4.1. Fragility Analysis

One area in which MCSs have been applied is fragility. Fragility is the vulnerability of a system to failure due to external or internal perturbations. It is inversely related to robustness [39], the capacity for a system to maintain its functions despite perturbations [40]. Prior to the use of MCSs for measuring structural fragility, EMs have been used to study the robustness of networks [41,42]; they have also been used in more recent studies on pathway knockout and redundancy in metabolic networks [43]. 

The application of MCSs to measure fragility can be found in [11,12,16]. The fragility coefficient, *F_i_*, defined as the reciprocal of the average size of all MCSs in which reaction *i* participates [12], is used as a quantitative measure for determining how essential the reactions are: the lowest value of *F_i_* would be closest to 0 where reaction *i* is one of many reactions occurring in a MCS, and the highest is 1 where reaction *i* is the only reaction in a MCS and therefore essential for the objective function. The average fragility over all the reactions is taken as the overall structural fragility of the network.

For example, in the network example *NetEx*, reaction R1 has two MCSs: the first MCS is MCS2 which has 2 reactions and the second is MCS6 which consists of 3 reactions; the fragility coefficient (*F_1_*) for R1 would therefore be 2/(2+3) which would be 2/5 or 0.4. The specific fragility coefficients of reactions in *NetEx* with respect to the production of P are as follows:

**Table 3:** Fragility coefficients of the reactions in *NetEx* with respect to the production of P.

	R1	R2	R3	R4	R5	R6	R7	*Psynth*
*Fi*	0.4	0.5	1	0.375	0.375	0.4	0.5	1

The above table shows that reaction R3 is essential for the production of *P* as is obviously the case for *Psynth*. This indicates that the loss of function of R3 would automatically render the other reactions meaningless for the production of *P*.

S. Klamt and E.D. Gilles [12] applied MCSs in their study of the central metabolic network of *E.coli*, earlier investigated by Stelling *et al* to study robustness using EMs. They found the number of MCSs to vary for different compound substrates that *E.coli* was growing on. For example, there were more MCSs, including the largest MCS for growth, on glucose than on acetate for which the lower number of MCSs were predominantly smaller. This indicated that *E.coli* growth on glucose was less fragile than on acetate. 

In the generalized MCS concept [11], Klamt further discussed their work on using MCSs to measure structural fragility of a network function. The results of the previous work [12] and other work [44,45] showed that environmental conditions, such as the type of substrates or availability of oxygen, greatly affected network properties like the essentiality of a gene/reaction, so it is important for a network structural fragility analysis to clearly define environmental conditions in addition to the deletion task describing the network functionality being considered.

### 4.2. Network Verification

MCSs can be used to verify a network because the minimal sets of target reactions/genes they provide are mathematically complete in relation to the structure of the network. Thus, the simultaneous removal of genes making up each MCS should lead to the elimination of the objective function. If the prediction is incorrect in an experiment and the phenotype is still viable, it means that the network structure is incorrect or incomplete. 

So, the set of MCSs could be systematically used to verify a given network structure by experimentally checking the phenotype predictions of MCSs in an organism: correct predictions would provide verification of the network whilst false predictions could be pursued to identify missing reactions/genes or compounds in the network structure. For example, say there is a reaction E = A + B in the network example *NetEx* (Figure 1) that has not been identified, applying MCS3 could reveal that there is a missing reaction in the network because compound E would still be formed and P synthesized.

Past work on network verification has been done using Flux Balance analysis (FBA) [46,47] and elementary mode analysis [42]. These were used to verify phenotype predictions for single mutants of *E.coli*, the predictions of which were found to highly agree with real mutants. In such cases the single mutation is lethal if the reaction involved is essential (a single reaction constituting a MCS) for the objective function, and depends on the chosen substrate. 

### 4.3. Observability of Reaction Rates in Metabolic Flux Analyses

Another use of the MCS concept is in finding the necessary information that can be used to make stationary network fluxes observable. As shown in [48], EM analysis (considering all reactions in the network as reversible) supports the identification of the set of known/measured flux measurements that would enable unknown non-measured reaction rates (*r_u_*) to be calculated or observed in a steady state flux distribution. The process includes first calculating all EMs and selecting those where the unknown reaction rate exists (*r_u_≠0*); the set of rates to be measured are then constructed such that they contain at least one of the reactions participating in each of the selected EMs. These sets of rates to be measured are in fact the MCSs with respect to the reaction rate (*r_u_* is the objective reaction) so, the sets of possible measurements will be minimal and non-redundant. As such, the MCSs can be screened to determine the most appropriate sets of measurement for FBA [49,50]. 

Take *NetEx* (Figure 1) as an example: considering all reactions as reversible and *r_u_* as *PSynth*, nine EMs are calculated, six of which lead to the synthesis of *P*. Taking *PSynth* as the objective reaction, there would be 10 MCSs for screening, as shown in Table 4 below:

**Table 4:** EMs and MCSs of *NetEx* (all reactions are reversible): A zero in an EM row indicates that the corresponding reaction is not involved in the EM corresponding to that row; a ‘1’ in a MCS row indicates that the reaction in that column constitutes the MCS corresponding to that row, e.g., R3 constitutes MCS1.

	R1	R2	R3	R4	R5	R6	R7	*PSynth*
EM1	1	0	1	-1	-1	1	0	1
EM2	0	1	0	1	1	0	0	0
EM3	1	0	0	0	1	1	-1	0
EM4	0	2	1	1	0	0	1	1
EM5	0	1	1	0	-1	0	1	1
EM6	1	1	1	0	0	1	0	1
EM7	0	0	1	-1	-2	0	1	1
EM8	1	-1	0	-1	0	1	-1	0
EM9	2	0	1	-1	0	2	-1	1
	R1	R2	R3	R4	R5	R6	R7	*PSynth*
MCS1			1					
MCS2								1
MCS3		1		1				
MCS4	1						1	
MCS5						1	1	
MCS6		1			1		1	
MCS7	1			1	1			
MCS8	1	1			1			
MCS9				1	1	1		
MCS10		1			1	1		

### 4.4. Pathway Energy Balance Constraints

To assist FBA, EMs have been used to place thermodynamics constraints at the pathway level [51] where a directionality criterion for net mass flux in the form of negative Gibbs free energy change (∆G) is applied to a pathway, as opposed to a reaction: the EMs matrix *E* is a *P*x*N* pathway composition, where *P* is the number of pathways. To formulate pathway level constraints *G*, the reaction-specific parameters are first collected into an *N*x*1* vector (∆g) and then an element-by-element multiplication is performed with each of the *P* (*N*-dimensional) rows of *E* to form *G*. The EMs ensure that the sequence of reactions in the entire pathway is in one direction and assist FBA in identifying the objective function(s) driving the metabolic behavior of tissue cells, especially multi-functional ones [51]. The relationship between MPA and FBA is discussed in detail in [52].

### 4.5. Target Identification and Metabolic Interventions

Along with their role in obtaining a deeper understanding of the structural fragility of cellular networks, MCSs can also be seen as minimal target sets for efficiently repressing cellular functions. The generalized concept [11] allows MCSs to tackle a larger variety of practical deletion problems, which include the repression of undesired metabolic functions, redirecting fluxes into a desired product, and inhibiting sub-optimal flux distributions. These in effect identify targets for metabolic interventions. 

For example, as illustrated in the intervention in Table 2 of *NetEx*, the set of MCSs (cMCSs) can be identified that would repress the synthesis of *P* and redirect fluxes to maximize the production of X. MCSs provide the capacity to identify an optimal intervention strategy by providing, from a structural perspective, the most efficient set of manipulations to achieve a certain deletion task. In addition to being efficient, an ideal MCS would be one that is small and therefore does not affect or weakly affect other network functions; also an MCS that does not involve network functions that are hard to eliminate e.g., a reaction with many isozymes.

The growing importance of MCSs in metabolic engineering is evident in [11,12,15], for example, [15] presents a model that uses MCSs to search for gene deletion strategies that would increase the production of microorganisms. In their approach, Hädicke and Klamt [15] address the limitation that MCSs have of disabling desired functionalities along with the targeted functionalities, by generalizing MCSs to cMCSs that allow for a set of desired modes, with a minimum number preserved, to be defined.

This generalization can be applied to existing methods which can be reformulated as special cMCS problems, providing the capacity for systematic enumeration of all equivalent gene deletion combinations and determining robust knockout strategies for coupled product and biomass synthesis, altogether offering great flexibility in defining and solving knock out problems. Other examples of MCSs in metabolic engineering can be seen in [14,29], discussed earlier in Section 3.2.

## 5. Similar concepts

### 5.1. Bottlenecks

Bottlenecks characterize a point of congestion in a system that happens when workloads arrive at a given point more quickly than can be handled at that point. In a metabolic network consisting of enzymes (nodes) and substrate-product metabolite fluxes (directional edges), three topological centralities that are used to measure the importance of nodes in controlling information transfer are: *in degree* which refers to the number of links forwarded to the node under consideration, *out degree* which refers to the number of links going out of the node, and *betweenness* which measures the number of “shortest paths” [53] going through the node. Bottlenecks are those nodes that have many “shortest paths” going through them, much like major bridges and tunnels on a highway map. 

**Figure 8:**
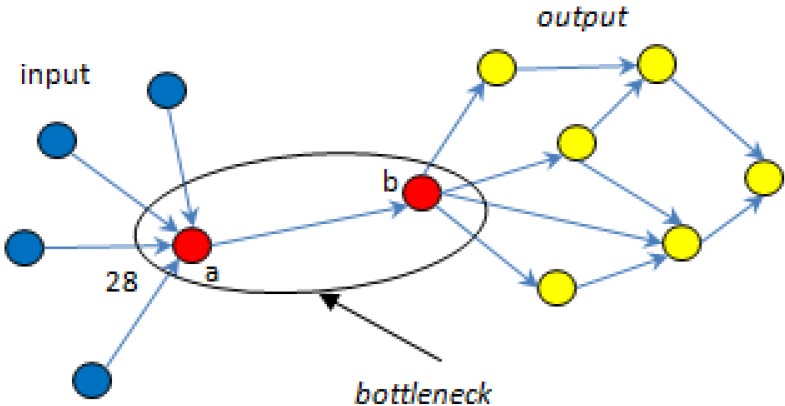
Example of a bottleneck in metabolic networks.

For example, the bottleneck nodes *a* and *b* in Figure 8 below, control most of the information flow because they form an essential highway to get information from the blue to the yellow nodes so, if either of nodes *a* or *b* is knocked out, the network would collapse. In effect, bottlenecks indicate essentiality of the nodes.

The essentiality of the bottleneck nodes is illustrated in the above graph which shows that they are “AND” nodes, traversed in series and you cannot get from the input nodes to the output except through node a “AND” node b. The *in degree* of node *a* is 4 and the *out degree* is 1; these centralities only consider the partners connected directly to a particular node, whereas the *betweenness* considers a node’s position in the network and, as shown for *a*, is much higher e.g. 28. Thus, bottlenecks in metabolic networks could be defined as nodes with a high *betweenness* centrality. 

One importance of bottlenecks is in relation to whole genome duplication (WGD) [54,55], where studies have shown that genes encoding hubs and bottleneck enzymes tend to express highly and evolve conservatively and thus were preferentially retained as homeologs [56]. Other studies include identification of novel targets for metabolic engineering of microorganisms used for sustainable production of fuels and chemicals [57] where the set of hub and bottleneck genes/enzymes were found to be a better strategy than manipulation of a single gene/enzyme.

In relation to MCSs, although MCSs can similarly determine the essentiality of enzymes, they do so in terms of repressing an objective function, represented by an objective reaction(s). For example, to use MCSs to calculate the essentiality of reactions/enzymes for a whole network, the objective function to repress would be the formation of all end products in the network, which would likely lead to combinatorial problems in larger networks. For the example network, *NetEx,* (refer to Figure 1), the objective reactions to repress in order to block all products are R5 and *PSynth*. In relation to the 6 EMs shown in Table 1, there are 16 MCSs for repressing the reactions R5 and *PSynth*. These MCSs are shown in Table 5 below with the corresponding fragility coefficients for each reaction:

**Table 5:** MCSs for *NetEx,* where all the EMs form the objective function. A “1” in the row of a MCS indicates inclusion of that reaction in the MCS, e.g, MCS1 consists of reactions R3 and R4, which means that simultaneous blocking of R3 and R4 would collapse *NetEx*. *Fj* shows the fragility coefficients of the reactions.

▪ MCSs	R1	R2	R3	R4	R5	R6	R7	*PSynth*	Total
▪ MCS1	0	0	1	1	0	0	0	0	2
▪ MCS2	0	0	1	0	1	0	0	0	2
▪ MCS3	0	1	1	0	0	0	0	0	2
▪ MCS4	0	1	0	1	0	0	0	0	2
▪ MCS5	0	1	0	0	1	0	0	0	2
▪ MCS6	0	0	0	1	0	0	0	1	2
▪ MCS7	0	0	0	0	1	0	0	1	2
▪ MCS8	0	1	0	0	0	0	0	1	2
▪ MCS9	1	0	0	0	1	0	1	0	3
▪ MCS10	1	0	0	1	1	0	0	0	3
▪ MCS11	1	0	0	1	0	0	1	0	3
▪ MCS12	1	1	0	0	0	0	1	0	3
▪ MCS13	0	0	0	0	1	1	1	0	3
▪ MCS14	0	0	0	1	1	1	0	0	3
▪ MCS15	0	0	0	1	0	1	1	0	3
▪ MCS16	0	1	0	0	0	1	1	0	3
▪ Total	4	6	3	7	7	4	6	3	
▪ *F_j_*	*0.33*	*0.43*	*0.5*	*0.39*	*0.39*	*0.33*	*0.33*	*0.50*	

The above table shows no reaction with a fragility coefficient [12] of 1, indicating that there is no essential reactions/enzymes (bottleneck) that, when blocked, would cause a collapse of the network *NetEx*. Bottlenecks would require a fragility coefficient of 1 because they represent an essential reaction that forms a bridge or tunnel to get from the input side of the network to the output. MCSs don’t necessarily have to, as shown by the fragility coefficients in Table 2 above, which can be used to extract information on the relative importance of reactions/enzymes. 

For example, ignoring the outermost reactions connected to the products (R5 and *PSynth*) in *NetEx*, R3 is the reaction with the highest fragility coefficient of 0.5. When we look at the corresponding EMs, R3 is also involved in the highest number of 5 EMs. Characterising that as a bottleneck does not seem unreasonable when looking at the *NetEx* diagram. In fact, adding the number of 1’s in the EM table is somewhat like the “*betweenness*” index that bottlenecks are based on. 

However, there is a significant difference: EM’s are not just shortest paths in the network; they are paths that are “short” in the sense of being irreducible, but their more important feature is that they cover all the mutually independent paths from substrates to products compatible with steady state. So, they reflect a lot more about the functioning of the network, not just the topology. Such *betweenness* in bottlenecks or derived from EMs, is basically what the fragility coefficient [12] expresses from MCSs. In effect, the fragility coefficient serves the same purpose as *betweenness* from the perspective of how fragile the structure of the network is at each reaction/enzyme but in a more comprehensive manner because it takes into account all MCSs that each reaction is involved in; in this respect the *betweenness* derived from MCS is much more informative for metabolism than the simple bottleneck concept.

### 5.2. Bow-Ties

The analysis of the connectivity structure of genome-based metabolic networks of 65 fully sequenced organisms [7] revealed that the global metabolic network was organized in the form of a bow-tie [7,58]. Metabolism has also been described as several nested bow-ties and large-scale organizational frameworks such as the bow-tie were necessary starting points for higher-resolution modeling of complex biological processes [59]. Studies and detailed information on the bow-tie topological features of metabolic networks and their functional significance can be seen in [7,58,59,60,61].

**Figure 9:**
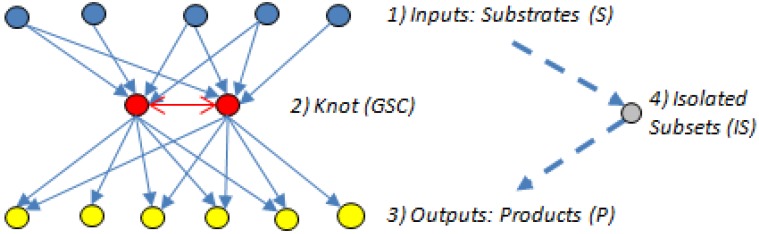
A simplified example of a bow tie.

The concept of bow-ties regards the metabolic network as a directed network. As illustrated in Figure 9 below, bow-ties [7,58,59,60,61], show similarity in structure to bottlenecks, except there is a difference in how the nodes are connected: the nodes that make up a bow-tie are “OR” nodes, *i.e.* they are traversed in parallel, while the nodes of a bottleneck are “AND” nodes, traversed in series. 

As illustrated above, the bow-tie structure of a directed graph has 4 components [7,58,59,60,61]:

(1) The input domain (substrate subset (S)), which contains substrates that can be converted reversibly to intermediates or directly to metabolites in the GSC, but those directly connected to the GSC cannot be produced from the GSC.
(2) The knot or GSC, which is the metabolite converting hub [60], where protocols manage, organize and process inputs, and from where, in turn, the outputs get propagated. The GSC follows the graph theory definition [62] and contains metabolites that have routes (can be several) connecting them to each other; it is the most important subnet in the bow-tie structure.
(3) The output domain (product subset (P)), which contains products from metabolites in the GSC and can also have intermediate metabolites but the products cannot be converted back into the GSC [7]. In other words, the reactions directly linking substrates to the GSC and the GSC to the products are irreversible.
(4) The resulting metabolites that are not in the GSC, S or P subsets form an isolated subset (IS), the simplest structured of the four bow-tie components [7], which can include metabolites from the input domain S or the output domain P but those metabolites cannot reach the GSC or be reached from it.

The bow-tie decomposition of a network can assist with the problem of combinatorial explosion encountered when calculating EMs and MCSs in large sized metabolic networks. For example, suppose that EMs are calculated separately for each of the three subnets (substrate S, GSC and product P), a typical EM for the full network can then be reconstituted by joining a substrate mode and product mode to one of their connecting GSC modes. The large number of ways in which this can be done is a manifestation of the combinatorial explosion, and demonstrates that the bow-tie splitting will substantially reduce the computational effort of calculating EMs and the resulting MCSs. 

More explicitly, the reactions constituting MCSs of a whole network can be classified in terms of the blocked reactions’ locations in the bow-tie decomposition:

(1) All substrate reactions (S subnet) plus GSC reactions blocking any cyclic EMs that could take place without inputs from the substrate reactions. In this case, no product reactions (P subnet) need blocking;
(2) All product reactions(P subnet) plus GSC reactions blocking the cyclic EMs- in this case no substrate (S subnet) need to be blocked;
(3) All GSC reactions that connect the S to the P subnet. No substrate or product reactions need to be blocked;
(4) A combination of S reactions plus GSC reactions reached from the unblocked S reactions. P reactions don’t need to be blocked;
(5) A combination of P reactions plus GSC reactions that could reach the unblocked P reactions. S reactions don’t need blocking.

These classifications can be used to investigate the question of whether a bow-tie decomposition can be derived from a known MCSs table. For example, a plausible strategy to identify GSC reactions is as follows:

- From all MCS, eliminate any that involve reactions that are known to belong to S or P;
- Order the remainder by increasing size and/or decreasing mean fragility coefficient;
- Choose a cutoff value in this sequence, and allocate all reactions that belong to MCSs in the top section of the sequence to the GSC.

If the bow-tie structure is pronounced, there should be a clear separation between the small, high fragility coefficient MCSs that belong to the GSC and the rest, otherwise the choice of a cutoff may be problematic. An MCS analysis may be helpful to examine if a bow-tie structure exists and partially detect members of its main components, but not to make a full partitioning.

Noting that bow-ties can assist with combinatorial explosion by decomposing large networks into subnets that can be analyzed by MCSs and EMs, we conclude that despite some overlap in the concepts and applications of bow-ties and MCSs, there is no clear cut correspondence between the two network descriptions. While bow-ties try to extract subsets of nodes that are of importance in the metabolic network, the EM and MCS approaches focus on comprehensive sets that are in different ways essential. Moreover, EMs are, by construction, the “constituents” of a steady metabolic state. So they, and MCSs, reflect the stoichiometry underlying the network and describe the metabolism, not just the topology of the network. In this respect, MCS (and EM) analysis is more powerful than bow-ties that just characterize network topology.

### 5.3. Weak Nutrient Sets

‘Weak nutrient sets’ is a concept analogous to MCSs, that was developed by Imielinski *et al.* [63] to demonstrate the duality between weak producibility and the existence of certain extreme semipositive conservation relations (ESCRs) in a media. ESCRs were defined as the simplest semi-positive linear combinations of species concentrations that were invariant to all metabolic flux configurations. A biochemical species was called producible in a constraints-based metabolic model if a feasible steady-state flux configuration existed that sustained its nonzero concentration during growth. 

Weak nutrient sets are analogous to MCSs in a metabolic network in that a MCS *C* for an objective reaction *j* is a set of reactions whose elimination renders flux through *j* infeasible at steady state so, a necessary and sufficient condition for *C* to be a cut set for *j* is that *C* is a hitting set for all *j*-containing elementary modes. Similarly, *U* is a weak nutrient set for species or metabolite *i* if and only if *U* is a hitting set for all of the *i*-containing ESCRs.

The ‘weak nutrient sets’ algorithm identified all minimal nutrient media that left an arbitrary metabolite weakly producible with respect to a given metabolic network. Details of the concept and its application can be seen in [63]. 

### 5.4. Flux Balance Analysis

Flux balance analysis (FBA) [49,64,65] shares a common underlying mathematical framework with MCSs and EMs except that, while EMs identify all possible and feasible non-decomposable metabolic routes for a given network at steady state, FBA derives a feasible set of steady-state fluxes optimizing a stated cellular objective e.g, optimizing the biomass production per substrate uptake. EM analysis establishes a link between structural analysis and metabolic flux analysis (MFA) where thermodynamically and stoichiometrically feasible stationary flux distributions for a network can be obtained from the linear combinations of the EMs. 

Calculating EMs and MCSs for larger networks can lead to problems with combinatorial explosion. However, because they are unique for a given network structure, they provide the full range of potential functionalities of the metabolic system and are therefore useful for investigating all physiological states that are meaningful for the cell in the long term. FBA, on the other hand, is more efficient, providing good predictions of mutant phenotypes and using linear programming to obtain a single (not necessarily unique) solution to an optimization problem. However, because it focuses on a specific behavior, FBA cannot cope with cellular regulation without additional constraints; it fails whenever network flexibility has to be taken into account, e.g., in the analysis of pathway redundancy or in quantitative prediction of gene expression [42].

We conclude that MCSs and EMs offer a convenient way of interpreting metabolic functions while FBA can be used to explore the relationship between the metabolic genotype and phenotype of organisms. MCSs, EMs and FBA can also be used together to interpret shifts in metabolic routing that could occur in response to environmental and internal/genetic challenges. Because they are mathematically equivalent, the predictions from the three methods would be the same except that MCSs enable the systematic search of more than one mutation.

## 6. Conclusions

MCSs are an extension of metabolic pathway analysis (MPA) methods and provide a way of identifying target genes for eliminating a certain objective function from a holistic perspective that takes into account the structure of the whole metabolic network and all the reactions taking place in the cell. The objective function can be represented by a set of EMs which is then used to calculate the MCSs used for studying structural fragility and identifying knock out strategies from a whole cell perspective. Such exhaustive characterization is very hard to achieve experimentally because, regardless of how many examples of a phenomenon one has observed, there might always be others not yet observed. This aspect of completeness by MCSs and EMs, subject only to a complete knowledge of the network itself, makes it possible to make quantitative assessments e.g. of the relative importance of reactions and their corresponding enzymes/genes. 

Looking in detail at the MCS concept and how it has developed in relation to similar concepts, it is easy to see its importance in systems biology and how it can contribute to fields such as metabolic engineering. Without needing prior knowledge of genes, MCSs can provide a complete list of loss of function(s) target genes that can then be investigated by other methods to analyze the properties of those genes and the impact that their suppression would have on other processes in the network. Thus, MCSs can assist in finding ways of producing industrially relevant compounds from renewable resources, not only for economical but also for sustainability reasons. 

The concept of MCSs is fairly new and still being explored; its similarity to other concepts and the fact that it has developed from the well established MPA method of EMs, means that MCSs can be used in conjunction with FBA and other MPA methods to get a better understanding of the capability of metabolic networks and the interrelationship between metabolites and enzymes/genes. The MCS concept also opens an avenue for developing new novel systems biology methods for use in genetic engineering. 
